# The Potential of Non-Ribosomal Peptide Engineering for Creating New Antimicrobial Complexes

**DOI:** 10.3390/molecules31040683

**Published:** 2026-02-16

**Authors:** Evgeniya V. Prazdnova, Maxim P. Kulikov, Ludmila E. Khmelevtsova

**Affiliations:** Academy of Biology and Medicine Named After D.I. Ivanovskiy, Southern Federal University, Stachki Avenue 194/1, Rostov-on-Don 344090, Russia; makkulikov@sfedu.ru (M.P.K.); lehmelevcova@sfedu.ru (L.E.K.)

**Keywords:** non-ribosomal peptides, self-assembly, nanostructures, antimicrobial peptides, NRPS engineering, antibiotic resistance, lipopeptides

## Abstract

Self-assembling antimicrobial complexes are a promising new technology for the development of antimicrobial, antifungal, and other bioactive agents with targeted delivery, adaptability, and the regulation of processes over time. Ribosomally synthesized antimicrobial peptides (AMPs) are most frequently considered as the basis for such complexes; however, we suggest that non-ribosomally synthesized peptides (NRPs) should be considered as molecules that also hold potential for engineering and already possess a set of qualities that AMPs are still to be engineered to have. This review examines the key features of NRP structure and self-assembly that determine their potential as antimicrobial agents, as well as NRP engineering methods through which new, more advanced agents for combating antibiotic-resistant microorganisms can be created.

## 1. Introduction

Non-ribosomally synthesized peptides (NRPs) are a broad class of compounds synthesized mainly by bacteria and fungi, with the latter apparently having acquired the genes for their synthesis via horizontal transfer [1]. NRPs are known for their antifungal and antimicrobial properties; however, they also possess a range of other biological activities that make them useful for biotechnology. A number of well-known antibiotics widely used in medicine and veterinary practice belong to this group of compounds (for example, polymyxin, bacitracin, actinomycin, vancomycin, gramicidin). Nevertheless, new compounds continue to be discovered every year, not least thanks to the evolutionary diversity of NR-synthesis genes.

The problem of growing antibiotic resistance forces scientists to search for ever newer compounds with activity against plant, animal, and human pathogens. In this search, the general line of the modern scientific agenda has turned to another group of compounds—antimicrobial peptides (AMPs). Unlike NRPs, they are synthesized ribosomally, are produced in practically the entire diversity of living organisms, and possess a number of properties that seem promising from the perspective of developing new drugs and molecular engineering [2,3]. This is, primarily, the ability to self-assemble into nanoscale supramolecular structures that are stable, adhere better to bacteria, penetrate target cells, and are protected from enzyme systems [4]. The capability for self-assembly and modification of such structures allows them to be considered a potential substrate for the development of targeted “smart drugs” that act in a specific place or at a specific time. Self-assembly refers to the spontaneous organization of individual molecular components into ordered supramolecular structures driven by non-covalent interactions, without external direction [5]. When applied to antimicrobial peptides, self-assembly creates nanostructures, including nanofibers, nanospheres, nanotubes, and hydrogels, that exhibit superior pharmaceutical performance compared to monomeric peptides. These nanocomposites demonstrate enhanced proteolytic resistance, prolonged systemic circulation, improved cellular internalization, and preferential accumulation at bacterial membranes [6].

However, it is rarely taken into account that non-ribosomal peptides are also capable of participating in such composite structures and have a tendency toward self-assembly. Furthermore, a number of their features endow them with some of the targeted beneficial properties. NRPS products, like daptomycin, surfactin, polymyxins, and tyrocidines, naturally incorporate features that synthetic AMP research aims to achieve: proteolytic stability due to incorporation of non-proteinogenic amino acids, stimuli-responsive assembly through metal ion coordination and optimized amphiphilicity through lipidation [7]. It was found that the NRP daptomycin is capable of overcoming bacterial resistance to AMPs caused by the action of a Bce-type efflux binding transporter. This makes it a promising candidate for clinical application [8].

But the difficulties of using existing NRPs in clinical practice, such as the high cost of synthesis, cytotoxicity, and the development of bacterial resistance, are also well known. In addition, new information regarding the behavior of self-assembling NRPs continues to emerge. Thus, it was recently discovered that the non-ribosomal lipopeptide colistin, produced by *Paenibacillus polymyxa*, contrary to popular belief, does not self-assemble but remains in a monomeric state under physiological conditions [9]. In this regard, it appears relevant to study the potential of NRPs for engineering new, more advanced variants, derived from the modular and domain structure of NRP synthases.

Self-assembling peptides, on which this review will be focused, are of great interest for practical use in the biomedical field. Clinical studies demonstrate that peptide hydrogels loaded with antimicrobial agents accelerate wound closure, reduce bacterial burden, and minimize scarring compared to conventional treatments. Hydrogels enable minimally invasive administration, forming protective antimicrobial barriers that conform to wound geometry [10]. For example, Feng et al. constructed a wound dressing based on a thermosensitive gel containing the self-assembling peptide RADA16 linked to an antibacterial peptide (AMP). Such a composite hydrogel possessed biocompatibility, good mechanical properties, as well as antibacterial and transport functions for wound healing [11].

Coatings based on antibacterial peptides can be used to prevent infection of catheters, implants, surgical instruments, etc. As is known, the biofouling of medical equipment by biofilms of microorganisms, often resistant to antimicrobial drugs, is a significant problem leading to serious postoperative complications and even the death of patients. Antimicrobial peptide coatings on catheters, implants, and surgical instruments prevent bacterial adhesion and biofilm formation [12]. Self-assembled peptide nanostructures provide durable, non-leaching coatings with sustained antimicrobial activity. The coatings can be designed to release antimicrobial agents in response to bacterial presence, providing on-demand protection. Another important biomedical application of self-assembling peptides is cancer therapy [13].

Encapsulation in nanoparticles can be an effective method for enhancing the therapeutic properties of self-assembling peptides, protecting the peptides from proteolysis, and ensuring targeted drug delivery due to the enhanced permeability and retention effect. Self-assembling nanocomposites with antimicrobial peptides can simultaneously deliver conventional antibiotics, creating synergistic combinations that overcome resistance mechanisms. Thus, the efficacy of a bacitracin–zinc nanodrug synthesized via zinc-coordinated self-assembly of bacitracin was demonstrated [14]. The nanodrug exhibited potent antibacterial action not only against Gram-positive *S. aureus* bacteria but also against *E. coli*. Alginate- and pectin-based nanoparticles combined with aggregated AMPs for dual antioxidant and antimicrobial action formed a stable delivery system, reducing inflammation and bacterial load 48 h post-infection [15].

In this review, non-ribosomal peptides are considered from an engineering perspective as building blocks for self-assembling antimicrobial complexes. To this end, the structural and functional diversity of non-ribosomal peptides that is directly related to self-assembly and membrane targeting is briefly described, and mechanisms are proposed for rationally reprogramming their modular and domain architecture to confer amphiphilicity, proteolytic stability and stimuli-responsive motifs that promote nanostructure formation. Finally, the review discusses how these engineered NRPs and NRP-inspired motifs can be integrated into self-assembling nanomaterials, including nanofibers, micelles, hydrogels and vesicles, to create next-generation antimicrobial and fungicidal complexes that address key challenges associated with antimicrobial resistance, toxicity and delivery.

In this regard, NRPs and their biosynthetic machineries can be viewed not only as sources of individual antibiotics, but as a versatile engineering platform for designing self-assembling antimicrobial complexes.

## 2. Comparison of NRPs with Representative Ribosomally Synthesized AMPs

Before focusing on the structural diversity, biosynthesis and engineering of non-ribosomal peptides, it is useful to first compare their key physicochemical and pharmacological properties with those of representative ribosomally synthesized human antimicrobial peptides. Such a comparison helps to clarify which features are unique to NRPs and why these molecules are particularly attractive as building blocks for self-assembling antimicrobial complexes. As representative ribosomal AMPs, it is logical to consider canonical human peptides such as LL-37 and HNP-1.

Structurally, LL-37 is a 37-amino-acid cationic, amphipathic α-helical peptide derived from the human cathelicidin precursor hCAP-18 [16]. HNP-1 is a 29–30-amino-acid α-defensin with a compact β-sheet structure stabilized by three disulfide bonds [17]. In contrast, many NRPs are cyclic or lipo-/glycopeptides that contain non-proteinogenic and D-amino acids, which confer high proteolytic stability and a pre-organized amphiphilic topology [18].

These peptides also differ in their antibacterial mechanisms. LL-37 disrupts membranes via a carpet/toroidal-type mechanism; its strong overall positive charge (net charge~+6) and amphiphilicity drive adsorption to bacterial membranes, defect formation, and in some cases, allow for translocation to intracellular targets [19]. HNP-1, by contrast, typically acts through a dimer-pore mechanism in which β-sheet dimers insert into the lipid bilayer to form cationic pores that dissipate ion gradients [20]. Many NRPs likewise induce membrane permeabilization, but often through more conformationally constrained architectures (macrocycles, lipid tails, rigid aromatic clusters) that favor stable pore formation or highly specific interactions with particular lipid species such as phosphatidylglycerol or LPS [21].

From a pharmacological standpoint, LL-37 and HNP-1 exhibit pronounced immunomodulatory activities, but their therapeutic use is limited by insufficient stability and host–cell toxicity, especially in systemic applications [22,23]. LL-37 is rapidly degraded in serum and wound exudate, and numerous reports describe cytotoxicity toward mammalian cells (for example, hemolysis and T-cell toxicity at low-to-mid micromolar concentrations), which has driven the development of shorter or cyclic derivatives with reduced toxicity and improved resistance to proteolysis [22]. HNP-1 is inherently more protease-resistant due to its disulfide-stabilized β-sheet structure; however, its antimicrobial activity and cytotoxicity can be attenuated by post-translational modifications such as ADP-ribosylation, which shift its function toward chemoattraction rather than direct killing [24]. Clinically used non-ribosomal peptides such as polymyxins and daptomycin, in turn, display high in vivo stability and well-defined pharmacokinetic profiles, but also illustrate that highly potent membrane-active NRPs can carry substantial risks of nephro-, neuro- or myotoxicity, thereby limiting the tolerated dose [25].

These differences are highly relevant for potential pharmacological combinations of NRPs with canonical human AMPs. Experimental studies on LL-37 and HNP-1 indicate context-dependent cooperative effects on membrane damage and epithelial barrier protection, suggesting that α-helical and β-sheet AMPs can modulate one another’s pore-forming behavior and impact on host tissues [26]. Conceptually, NRPs could either act synergistically with LL-37 or HNP-1, reducing the required NRP dose (and thus its toxicity) while enhancing targeted membrane disruption, or conversely exacerbate host–cell damage and inflammatory responses through cumulative increases in membrane permeability. Systematic studies that analyze synergy or antagonism between specific NRPs and LL-37/HNP-1 remain scarce. Nevertheless, the described features of their membrane activity, proteolytic stability and immunomodulatory profiles provide a rational basis for predicting the therapeutic potential and safety of such combinations. Table 1 briefly summarizes the key comparative properties of canonical ribosomal and non-ribosomal peptides.

Taken together, these comparisons underscore that non-ribosomal peptides occupy a distinct region of chemical and pharmacological space compared to canonical human AMPs, combining high structural complexity, proteolytic stability and potent membrane activity with both therapeutic potential and toxicity risks. In the following sections, the focus therefore shifts specifically to NRPs, examining their diversity, unique structural features, biosynthetic mechanisms and engineering strategies that can be leveraged to construct self-assembling antimicrobial complexes.

## 3. The Variety of NRPs in Bacteria and Fungi

There are several approaches to the classification of NRPs. By origin, NRPs can be divided into pro- and eukaryotic, or more precisely, bacterial and fungal, since these organisms are their main producers. Among prokaryotes, the most active NRP producers belong to the *Bacillus* [27,28], *Pseudomonas* [29], *Streptomyces* [30] genera, and cyanobacteria groups [31]; among fungi, representatives of the genera *Aspergillus* [32] and *Trichoderma* [33]. In rare, isolated cases, more complex eukaryotic organisms also act as NRP producers; proven cases in this category are the NRPS “Ebony” from the fruit fly *Drosophila melanogaster* [34] and nemamide synthetase from the nematode *Caenorhabditis elegans* [35].

Based on their spatial structure, cyclic NRPs are divided by ring size (e.g., gramicidin S, cyclosporine), including lipocyclopeptides having additional linked acyl groups (e.g., surfactin, iturin, fengycin). Linear peptide structures are also distinguished, varying from tripeptides (e.g., sevadicin, bialaphos) to 20-mer peptides (peptaibols, e.g., alamethicin), and the upper limit for the number of amino acids in linear NRPs is approximately 25 amino acid residues [36].

NRPs can also be classified based on their biological activity. In particular, antibacterial (daptomycin, vancomycin, colistin, polymyxin, and many others) [37], fungicidal (surfactin, fengycin) [28], cytostatic (bleomycin) [38] and immunosuppressive peptides (cyclosporine A) [39], as well as siderophores (enterobactin, bacillibactin, vibriobactin, yersiniabactin) [40].

It is also possible to classify NRPs based on the structure of their synthases (NRPSs), since their structural diversity is determined primarily by the features of the NRPS architecture. Most often, NRPSs are divided into two main classes. Type I NRPSs are large modular complexes containing all the enzymes necessary to generate the peptide product as a single assembly line. Type II NRPSs contain autonomous enzymes or didomains that coordinate to form peptide products. Often, they are represented by individual A- and PCP-domains, as well as their combination. This class of NRPSs was first described for bleomycin [19].

Hybrid NRPS-PKS systems are also known [41]. Such systems have their own variety of ways of combining NRPS and PKS modules. Such hybrid systems formed into a single multimodular structure, where NRPS and PKS modules are combined and may contain modules of different types for PKS, are often present in microorganisms. An example is polyene antibiotic bacillaene synthetase [42]. The second type of hybrid systems is known primarily in fungi, where one iterative PKS module (including several acetate building blocks) is followed by one NRPS module (fusion of the polyketide chain with an amino acid) [43]. Such architecture was shown for the synthesis of pretenellin A [44].

Another unusual group of secondary metabolite synthases producing dipeptides with a wide spectrum of biological activity is worth highlighting separately—cyclodipeptide synthases, which carry out the synthesis of diketopiperazine compounds [45]. This group of secondary metabolites is particularly interesting due to the presence of fungicidal activity in its representatives. Thus, in particular, cyclo(L-Phe-L-Pro) and cyclo(L-Phe-trans-4-OH-L-Pro) have antifungal properties [46], and bicyclomycin is a substance with antibacterial activity [47]. Among them are compounds with antitumor activity (ambevelamides A and B), as well as antiviral and immunosuppressive activities (gliotoxin, sirodesmin) [48]. There are also specific features in the methods of diketopiperazine biosynthesis. NRPSs can be directly responsible for their synthesis, producing them as target products (thaxtomin A, gliotoxin), or they can arise as by-products during the non-ribosomal synthesis of longer peptides. This occurs, in particular, during the synthesis of tyrocidine A, in which cyclo(D-Phe-L-Pro) is released [49].

## 4. NRPs Unique Structures and Properties

Non-ribosomal peptides possess unique structural properties including over 500 non-proteinogenic monomers (D-amino acids, modified residues), extensive N-methylation and lipidation, cyclic/polycyclic architectures with oxazoline/thiazoline heterocycles, and post-assembly modifications (glycosylation, acylation, halogenation, hydroxylation) that collectively provide proteolytic resistance, amphiphilicity, and self-assembly ability, sometimes exceeding ribosomally synthesized peptides [50,51,52]. The most remarkable feature of NRPs is that they include non-canonical monomers that are unavailable in ribosomal synthesis. These include D-amino acids (created through epimerization domains that convert L-amino acids to their D-enantiomers), non-proteinogenic amino acids such as 2-aminoisobutyric acid (Aib), ornithine (Orn), 2,4-diaminobutyric acid (Dab), hydroxyphenylglycine (Hpg), and β-amino acids [53,54,55]. The presence of D-amino acids is particularly significant: these stereoisomers play crucial roles in determining peptide conformation, enhancing biological activity, and, most importantly, conferring resistance to proteolytic degradation by limiting recognition by proteases that evolved to cleave L-amino acid bonds [56].

Additionally, NRPSs incorporate significant post-assembly modifications including N-methylation (which increases lipophilicity and oral bioavailability while stabilizing conformations), N-formylation, glycosylation, acylation, halogenation, and hydroxylation. N-methylation of backbone amide groups eliminates hydrogen bond donors, disrupting protease recognition and dramatically extending serum half-lives, as exemplified by cyclosporin A, where multiple N-methylations contribute to exceptional proteolytic stability and therapeutic efficacy [57].

The primary structural architecture of NRPs deviates radically from linear peptide chains: nearly 75% of characterized NRPs possess nonlinear structures including full or partial cyclization, branching, or complex polycyclic frameworks. Cyclization occurs through various mechanisms—backbone-to-backbone (head-to-tail), backbone-to-sidechain (forming lactones or lactams), or sidechain-to-sidechain connections—often catalyzed by thioesterase (TE) domains or cyclization (Cy) domains during peptide assembly [58]. The cyclic constraint reduces conformational entropy of the unfolded state, thereby stabilizing the folded bioactive conformation and increasing the free energy barrier against proteolytic degradation. For instance, tyrocidines and gramicidin S are cyclic decapeptides that form rigid ring structures incapable of fitting into protease active sites, conferring remarkable stability [59]. Serine, threonine, and cysteine residues—highly frequent in NRPs—enable the formation of oxazoline and thiazoline heterocycles through Cy domain-catalyzed cyclodehydration, creating five-membered rings that further rigidify structures and resist enzymatic cleavage [60]. Glycopeptide antibiotics like vancomycin exemplify extreme structural complexity: the linear heptapeptide scaffold undergoes oxidative crosslinking by cytochrome P450 enzymes, creating three aryl ether bridges and two additional biaryl linkages that lock the molecule into a rigid cup-shaped architecture specifically evolved to bind D-Ala-D-Ala termini of bacterial peptidoglycan precursors [61].

The unique structural features of non-ribosomal peptides—particularly lipidation, cyclization, and incorporation of non-proteinogenic amino acids—make them excellent building blocks for self-assembling antimicrobial nanostructures. Lipidation provides amphiphilicity that drives spontaneous self-assembly into micellar, vesicular, and fibrillar nanostructures above critical aggregation concentrations (CAC) [62]. It was demonstrated that daptomycin forms spherical micelles of 14–16 monomers, while surfactin assembles into elongated direct micelles in aqueous solutions [63,64]. These naturally occurring lipopeptide nanostructures exhibit concentration-dependent morphological transitions, from isolated molecules to spherical micelles to cylindrical micelles or vesicles, controlled by packing parameters determined by lipid tail length, peptide charge, and environmental conditions (pH, ionic strength, temperature) [65].

The cyclic nature of many NRPs provides conformational rigidity that stabilizes assembled structures while simultaneously conferring exceptional proteolytic resistance: cyclic peptides maintain bioactivity even after prolonged exposure to serum proteases, with half-lives exceeding 48–168 h compared to several minutes for linear peptides [45]. Furthermore, the incorporation of D-amino acids, β-amino acids, and N-methylated residues creates peptide backbones that resist enzymatic degradation while maintaining self-assembly propensity—enabling the design of protease-resistant self-assembling antimicrobial systems that combine the therapeutic advantages of both structural classes [66]. The multivalent presentation of antimicrobial peptide motifs on nanostructure surfaces dramatically enhances bacterial membrane binding through cooperative effects, while structural constraints within assembled architectures can mask cytotoxic hydrophobic residues, improving selectivity for bacterial over mammalian membranes.

These properties position NRP-inspired design principles—lipidation for amphiphilicity, cyclization for stability, and non-canonical modifications for protease resistance—as a potential foundation for next-generation self-assembling antimicrobial peptide nanocomposites that overcome the major limitations (proteolytic degradation, cytotoxicity, poor pharmacokinetics) of conventional antimicrobial peptides.

## 5. The Range of NRP Bioactivity

Due to their extraordinary structural diversity, NRPs possess a broad spectrum of biological activity, ranging from siderophores and biosurfactants to immunomodulators, cytotoxins, and neuroprotective agents.

Primarily, NRPs have become known for their broad, often non-specific antibacterial activity that applies even against multi-resistant strains. For example, delftibactin A is antagonistic to both Gram-positive (MRSA and VRE) and Gram-negative (*Acinetobacter baumannii*) multi-resistant bacteria [67]. An equally important aspect is the antifungal activity of non-ribosomal peptides. Lipopeptides produced by various *Bacillus* species, such as bacillomycin and iturin [68,69], surfactin [70,71] and fengycin [72,73], are known for antifungal activity against various phytopathogens.

An extremely diverse group of NRPs are siderophores, synthesized by many groups of bacteria and fungi. Siderophores are exported into the environment under low iron levels and reimported as Fe^3+^ complexes to provide iron for cellular processes. Prominent representatives are enterobactin of *E. coli* [74], salmochelin of *Salmonella* [75], bacillibactin of *B. subtilis* [76], pyoverdine of *Pseudomonas* [77], and mycobactin of *Mycobacterium* [78].

Among the most clinically significant activities are their immunosuppressive capabilities. Cyclosporin A, which inhibits T-cell activation by binding to cyclophilin and blocking calcineurin-mediated dephosphorylation of the nuclear factor of activated T cells (NFAT), thereby preventing lymphokine gene transcription [79]. Similarly, rapamycin (sirolimus), biosynthesized through a hybrid polyketide synthase–NRPS system, functions as a potent immunosuppressant by inhibiting the mammalian target of the rapamycin (mTOR) pathway, blocking IL-2 and other cytokine receptor-dependent signal transduction mechanisms [80].

Beyond immunosuppression, NRPs demonstrate remarkable antitumor and anticancer activities [81], with bleomycin serving as a prominent example that binds to guanosine-cytosine-rich DNA regions and causes oxidative DNA damage through iron-mediated mechanisms, making it clinically valuable for treating testicular cancer, Hodgkin’s disease, and other malignancies [82]. The anticancer potential extends to compounds like dactinomycin (actinomycin D), which intercalates DNA and inhibits RNA polymerase-mediated transcription, inducing immunogenic cell death that sensitizes tumors to subsequent immunotherapy [83].

The ionophoric activities of certain NRPs enable sophisticated ion transport mechanisms, as demonstrated by valinomycin, which forms selective complexes with potassium ions and facilitates their transport across lipid membranes with remarkable specificity (100,000-fold preference for K+ over Na+), disrupting cellular ion homeostasis [84]. Polymyxins exemplify membrane-targeting NRPs that bind to lipopolysaccharide (LPS) pyrophosphate groups in bacterial outer membranes through their polycationic structure, causing membrane depolarization and permeabilization, though their clinical utility is limited by toxicity concerns [85].

Additional bioactivities of non-ribosomal peptides include enzyme inhibition, toxin production, and modulation of cellular signaling pathways.

It is worth noting a group of NRPs that represent toxins for humans and animals. For example, microcystins, cyanobacterial hepatotoxins, potently inhibit serine/threonine protein phosphatases PP1 and PP2A by covalently binding to cysteine residues in the catalytic subunit, leading to hyperphosphorylation of cellular proteins and cytoskeletal disruption, while also protecting the producing organism against oxidative stress through protein stabilization [86,87]. Although the physiological role of toxins for the producing organism is not always clear, the consequences of entering the animal or human body can be both acute and chronic, and vary from irritant, allergenic, neurotoxic, or hepatotoxic to carcinogenic and mutagenic effects. Toxins of these groups include diketopiperazine-type peptides, for example, chaetomin, gliotoxin, roquefortine, verruculogen, and fumitremorgin A [88]. Enniatins, which are cyclic hexadepsipeptide mycotoxins, exhibit ionophoric properties that disrupt intracellular cation concentrations, inhibit acyl-CoA cholesterol acyltransferase (ACAT), induce mitochondrial dysfunction, and demonstrate insecticidal, antifungal, antibacterial, and potential anticancer activities through mechanisms involving oxidative stress and apoptosis induction [88].

Cell wall biosynthesis inhibitors include vancomycin, which forms hydrogen-bonded complexes with the D-alanyl-D-alanine termini of peptidoglycan precursors, preventing their incorporation into the growing cell wall [89], and the echinocandins, which noncompetitively inhibit β-1,3-glucan synthase in fungal cell walls [90].

Specialized membrane activities are exemplified by gramicidin A, which forms ion channels in lipid bilayers through dimerization, selectively conducting cations across membranes, and daptomycin, which binds to lipid II and phosphatidylglycerol in bacterial membranes in a calcium-dependent manner, causing membrane depolarization and cell death [91].

## 6. Mechanisms of NRP Synthesis

The biosynthesis of non-ribosomal peptides is carried out by NRPSs, which are modularly organized multienzyme complexes. A module is defined as a part of the multienzyme complex that specifically incorporates one amino acid into the peptide backbone, and, in turn, modules can be divided into domains that catalyze individual steps of non-ribosomal peptide synthesis. Each module consists of at least three domains: an adenylation domain (A), a peptidyl carrier protein (PCP) or thiolation (T) domain, and a condensation domain (C) [92]. The order of modules is usually collinear with the sequences of the peptide products [93].

The synthesis of non-ribosomal peptides proceeds in the N-to-C terminal direction, forming peptides that are usually about 3–15 amino acids in length, and the released peptides can be linear, cyclic, or branched-cyclic. Furthermore, in some cases, the presence of so-called secondary domains is possible, performing modifications such as epimerization, methylation, or formylation, which, as it was stated above, increase stability of these secondary metabolites [93,94].

The first step in biosynthesis is performed by the A-domain, which recognizes and performs the activation of the amino acid substrate via adenylation using Mg-ATP, resulting in the formation of an aminoacyl-adenylated intermediate [95]. The A-domain consists of ~550 amino acids and has ten amino acid residues that can be viewed as “codons” of NRPS enzymes and are important for substrate specificity [96]. Substrates that can be recognized by the A-domain may include the D- and L-forms of the 20 amino acids used in ribosomal protein synthesis, as well as non-proteinogenic amino acids such as ornithine and hydroxy acids, such as α-aminoadipic and β-butyric acids [94].

The second step is performed by the peptidyl carrier protein domain (PCP-domain), consisting of approximately 80 amino acids, which covalently binds the activated amino acid to its cofactor 4′-phosphopantetheine (PP) via a thioester bond and transfers the activated substrate and elongation intermediates to the C-domain [97].

The final step is performed by the C-domain of ~450 amino acids, which catalyzes the formation of a peptide bond between the carboxyl group of the nascent peptide and the amino acid carried by the flanking module, allowing for the translocation of the growing chain to the next module [82]. After the condensation step, the linear intermediate peptide is released by the thioesterase (TE) domain via hydrolysis or internal cyclization in bacteria, and less frequently, in fungal NRPS [98].

## 7. Approaches to NRP Engineering

The modular principle of NRPS organization provides the opportunity for engineering interventions to obtain new diverse NRPs, including new antibiotics and other bioactive natural products that are too complex for chemical synthesis.

An engineering can be performed both at the level of gene sequences encoding NRPS modules and at the level of domains (or entire modules) through their replacement, deletion, rearrangement, modification, etc. In recent years, the development of evolution-inspired recombination frameworks, synthetic type S NRPS architectures and CRISPR-based genome editing tools has expanded the engineering toolbox for non-ribosomal peptide biosynthesis, moving the field from single-enzyme modifications toward the systematic redesign of entire assembly lines.

### 7.1. Domain Engineering

NRPS modules can accommodate integrated tailoring domains that modify peptide intermediates during assembly. These include epimerization (E), methylation (MT), cyclization (Cy), oxidation (Ox), and reductase (R) domains that act on peptidyl carrier protein (PCP)-tethered substrates [99].

Natural recombination patterns provide valuable information for the development of engineering strategies. A comprehensive analysis of NRPS evolution shows that recombination is a key factor in the diversification of bacterial NRPS. Notably, recombination mainly affects only adenylation (A) domains, causing partial substitutions in the A core subdomain without requiring the co-evolution of adenylation and condensation domains, as previously thought [100].

#### 7.1.1. A-Domain

Historically, the first experiments on NRPS engineering focused on domain substitutions. In one of the first studies, Stachelhaus et al. constructed a hybrid surfactin synthetase with altered specificity to the amino acid substrate by replacing the A-domain recognizing Leu in SrfA-C from *B. subtilis* with an A-domain recognizing Cys [101]. In 2020, Calcott et al. obtained modified pyoverdine peptides with high yield by replacing only A-domains [102]. Domain modification can also be an effective tool for obtaining new products. Of particular interest in this regard is the aforementioned adenylation domain A, being a key catalytic domain that primarily controls the product sequence during the NRP assembly process and, thus, plays a defining role in NRP structural diversity [103].

Various modification strategies often target adenylation domains to alter substrate specificity. These include mutagenesis of substrate specificity codes, replacement of condensation-adenylation didomains, complete A-domain replacement, domain insertion, and rearrangement of entire modules. Subdomain replacement strategies have proven particularly effective, using flavodoxin-like subdomains involved in substrate recognition [104].

Taken together, these studies indicate that adenylation domains can be retuned with comparatively minor sequence changes or subdomain swaps, making them primary targets for rationally redirecting NRP building block selection in engineered assembly lines.

#### 7.1.2. C-Domain

Given that the C domain, which catalyzes the formation of peptide bonds between substrates, exerts a certain influence on substrate selection specificity by the A domain, a number of studies have focused on it. Modifications of condensation domains were traditionally considered difficult due to the presumed strict specificity to the acceptor substrate. However, recent data suggest that C-domain substrate specificity may be less strict than previously thought. High-throughput approaches to engineering C-domains responsible for peptide elongation have demonstrated successful reprogramming for binding non-native substrates [105].

#### 7.1.3. TE-Domain

Thioesterase domains not only release products but can be repurposed to control ring size, topology and even introduce additional transformations such as epimerization. Engineering studies on TycC and NocTE have demonstrated broad substrate tolerance and dual catalytic roles, while TEII enzymes such as GrsT can significantly boost productivity by clearing stalled intermediates. Together, these features make TE domains powerful levers for tuning macrocyclization and yield within engineered NRPS systems [106,107,108].

#### 7.1.4. E-Domain

Epimerization domain engineering has proven particularly successful for controlling stereochemistry. E domains catalyze L → D amino acid conversion and can be relocated between modules to introduce D-amino acids at different positions. Using tyrocidine synthetase A as an example, it was shown that the E-domain of TycA, which usually epimerizes phenylalanine, is also capable of accepting alternative substrates—tryptophan, isoleucine, and valine—albeit with lower efficiency, and also that the epimerization activity is influenced by the nature of amino acid residues located near the Ppant cofactor binding site [109].

#### 7.1.5. Cy-Domain

The heterocyclization (Cy) domain represents a specialized condensation domain that performs dual functions. Unlike standard C domains with the HHxxxDG motif, Cy domains contain a DxxxxD motif and catalyze both peptide bond formation and subsequent heterocyclization of Cys, Ser, or Thr residues to form thiazoline, oxazoline, or methyloxazoline rings. These domains can be engineered to introduce heterocyclic moieties into non-native peptide contexts [52].

#### 7.1.6. PCP (Peptidyl Carrier Protein) Domain

The peptidyl carrier protein (PCP) domain acts as a central hub in NRPS assembly lines, transiently binding peptide intermediates and mediating their transfer between adenylation, condensation and tailoring domains [110]. As a consequence, PCP–partner interfaces are important determinants of whether domains and modules remain compatible when NRPSs are recombined or redesigned. Structural and mutational studies have shown that modest changes in exposed surface regions, such as loop 1 and adjacent helices, can alter recognition of cognate A or C domains and rescue activity in otherwise poorly functioning chimeric systems [111]. Thus, PCP domains should be regarded not only as passive carriers, but also as key engineering targets for tuning interdomain communication and ensuring efficient peptide transfer in synthetic or hybrid NRPS architectures.

#### 7.1.7. Cs and AL Domain Engineering

Starter condensation (Cs) and acyl ligase (AL) domains determine which fatty acid or other acyl group is attached at the beginning of lipopeptide biosynthesis, and thus strongly influence hydrophobicity, membrane affinity and self-assembly behavior [112]. Mutations in the substrate-binding pocket of AL domains can shift chain-length specificity, while the FAAL-like activation mechanism provides a clear structural basis for rational design of these preferences. Beyond natural fatty acids, engineering of starter units has been extended to aromatic carboxylic acids, heterocycles and modified fatty acid analogs, generating lipopeptide libraries with systematically varied lipid moieties for structure–activity studies [113].

In practice, tuning Cs/AL domain specificity and starter unit composition offers a direct route to modulate the amphiphilicity and membrane interactions of NRP-based lipopeptides, which is highly relevant for designing self-assembling antimicrobial complexes [114].

#### 7.1.8. Tailoring Enzyme Engineering

Separately encoded tailoring enzymes can be engineered to interact with NRPS systems. These include halogenases, oxidases, hydroxylases, and cytochrome P450 enzymes that act non-covalently on PCP-tethered intermediates. The protein-protein interfaces between PCPs and tailoring enzymes provide specific targets for engineering enhanced or altered substrate recognition [115]. Research has revealed the structural basis for these interactions, showing that tailoring domains recognize specific motifs on carrier proteins. This understanding enables rational engineering of both the carrier protein and tailoring enzyme interfaces to expand substrate scope or introduce new chemical modifications [116].

### 7.2. Module Engineering

Beyond domain manipulations, NRPS engineering can be performed at the level of entire modules, including their rearrangement, replacement, deletion, or, conversely, addition.

The modular architecture of NRPS systems provides natural breakpoints for engineering interventions. Studies have demonstrated successful rearrangements of entire modules using several approaches. Module replacement was implemented in daptomycin synthetase, where researchers swapped modules 8 and 11 (responsible for recognizing D-Ala and D-Ser, respectively), demonstrating the feasibility of such an approach despite a reduction in yield compared to the original strain [103].

Module deletion represents another effective strategy. It was first described over two decades ago, for example, in the work of Mootz et al., who successfully removed the SrfA-A2 module from the surfactin NRPS assembly line by directly connecting modules 1 and 3, resulting in the conversion of the original heptapeptide into a hexapeptide variant (Δ2-surfactin). Furthermore, it was found that module replacement resulted in significantly higher product yields than observed in previous studies [117]. Module deletion and insertion have been used to design complexes capable of producing new lactones. Thus, the authors succeeded in contracting the tetralactone ring of neoantimycin A by deleting the NatD NRPS module from the nat cluster (yielding a trilactone), as well as expanding the trilactone JBIR-06 (2) to a tetralactone by attaching the NatD NRPS module to the sml cluster [118].

Modification of NRPS gene clusters is often difficult due to both their large size and the presence of repeats (as seen, for example, in plipastatin, a cyclic decapeptide of *B. subtilis*). Jagadeesh and colleagues proposed an approach called the “Seam Express Assembly Method (SEAM)”, combined with Ordered Gene Assembly in *B. subtilis* (OGAB), which involves introducing restriction sites as seams at module boundaries. The application of SEAM–OGAB for chimeric assembly was demonstrated by swapping plipastatin NRPS and surfactin NRPS modules to generate two new lipopeptides in *B. subtilis*. The advantage of the SEAM–OGAB method is that it easily facilitates the shuffling of NRPS gene modules for the production of new peptides [119].

In a later study by the authors, the SEAM-OGAB approach was combined with the combi-OGAB method to construct combinatorial DNA libraries of plipastatin and gramicidin S NRPS genes for the random replacement of NRPS module fragments. The researchers obtained 32 types of plasmid DNA from 50 randomly selected transformants, yielding at least 30 types of peptides ranging from 5 to 10 amino acids in length [120]. An improvement to the combi-OGAB method was proposed by Miyamoto et al. [121]. They developed a directed evolution method that combines random mutagenesis via error-prone PCR and Combi-OGAB (rm Combi-OGAB). Plasmids producing gramicidin S, initially obtained using conventional Combi-OGAB, were modified using rm Combi-OGAB. Notably, *B. subtilis* carrying the evolved plasmid with unpredictable mutations demonstrated a 1.5-fold increase in gramicidin S production. The SEAM-combi-OGAB method represents a breakthrough in creating large combinatorial libraries of NRPS gene clusters. Nevertheless, the success of such strategies is often limited. Exchanging subunits or modules can disrupt interdomain contacts, reducing product yields or activity.

**Figure 1:**
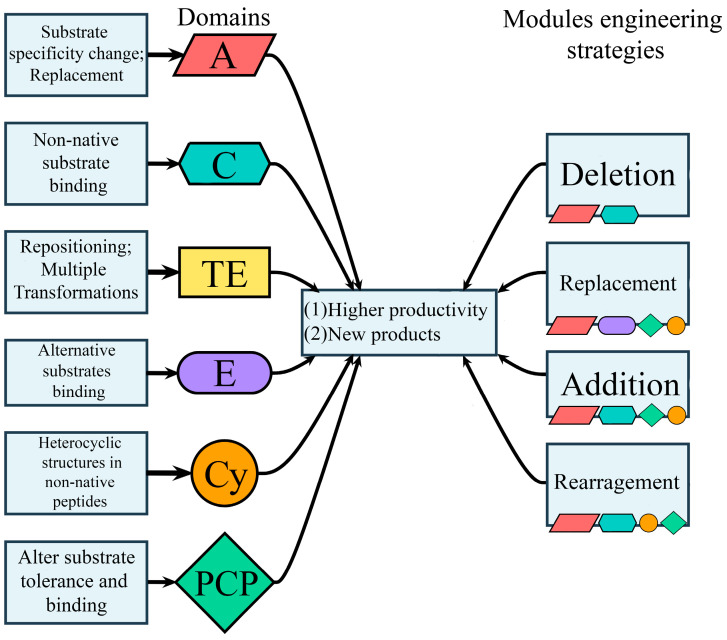
NRP Engineering Strategies.

The main approaches to engineering domains and modules of the NRP are summarized in Figure 1.

### 7.3. NRPS Gene Editing

Due to the large size and structural complexity of NRPS gene clusters, they are difficult to manipulate using traditional genetic engineering methods. However, the development of modern tools, such as CRISPR-Cas9, offers a good opportunity for editing NRPS gene sequences. Hou et al. presented practical editing tools allowing for the effective knockout of entire NRPS/PKS gene clusters; a strategy for genome splitting and plasmid capture of NRPS/PKS genes was also described. The competing biosynthetic gene cluster of the lipopeptide iturin A in *B. amyloliquefaciens* was knocked out, and the NRPS/PKS gene cluster responsible for its production was transferred to a plasmid. The constructed strain demonstrated significantly increased gene expression and increased production of iturin A [122].

CRISPR-Cas9 gene editing is being increasingly applied to NRPS engineering, enabling precise modifications of biosynthetic gene clusters. This approach facilitates rapid construction of variant NRPS systems and enables multiplexed engineering approaches [123].

Regulatory genes with pleiotropic effects, the expression of which determines the synthesis of a whole set of NRPs, can also become targets for gene editing, aimed at enhancing secondary metabolite production. In *Streptomyces* species, pleiotropic regulators such as *adpA*, *bldA*, and *absB* exert multi-layered control over antibiotic biosynthesis, often bypassing cluster-situated regulators (CSRs) to directly govern structural gene expression [124]. Manipulation of *adpA* expression under a strong constitutive promoter resulted in a 2.5-fold increase in moenomycin production, validating this regulatory gene as an effective engineering target.

Similarly, in *Burkholderia thailandensis*, the LysR-type transcriptional regulator ScmR functions as a master gatekeeper that pleiotropically represses multiple BGCs including those encoding non-ribosomal peptides; deletion of *scmR* resulted in hyperactive secondary metabolite production with dramatic increases in malleilactone, burkholdac, and other compounds [125]. These global regulators often operate through quorum sensing (QS) circuits, as demonstrated in *Xenorhabdus szentirmaii*, where quorum sensing genes synchronize the expression of 17 distinct NRPS/PKS operons responsible for antimicrobial compound synthesis [126].

Recent advances in CRISPR-based technologies have revolutionized the engineering of pleiotropic regulatory genes for NRP production optimization. Yan et al. developed a screening-based multi-target rational combination strategy using CRISPR interference (CRISPRi) to identify genome-wide repressors inhibiting NRP biosynthesis, successfully enhancing daptomycin, thaxtomin A, and surfactin titers by 17–38% through targeted knockdown of transcriptional repressors like *wblA* and *tetR*6 [127]. This approach addresses the challenge that 12% of *Streptomyces* genomes encode regulatory genes whose intricate networks evolved for survival benefits rather than yield optimization. Ameruoso demonstrated that CRISPRi targeting of the repressor *jadR2* synthetically activated the silent jadomycin B biosynthetic gene cluster in *Streptomyces venezuelae*, while CRISPRa (CRISPR activation) using dCas9 fused to transcriptional activation domains enabled direct upregulation of BGC expression with up to 20-fold increases when targeted at optimal promoter positions exhibiting 10 bp periodicity patterns [128]. Zhou further expanded this toolkit by repurposing endogenous type I-E CRISPR-Cas systems in non-model *Streptomyces* strains, successfully activating 13 cryptic BGCs including NRPS-encoding clusters and enabling discovery of novel linaridins and polyketides [129]. Augustijn demonstrated that SARP (Streptomyces Antibiotic Regulatory Protein) family regulators—particularly small SARP subtypes—serve as genomic beacons for discovering hidden NRPS BGCs, with 82 previously undetected BGCs identified through systematic analysis of regulatory gene-BGC associations across 440 complete *Streptomyces* genomes [130].

Thus, multiplex CRISPR–Cas platforms capable of editing long NRPS/PKS clusters and associated regulatory networks open the door to holistic pathway rewiring rather than isolated gene changes. In combination with computational design and machine-learning-guided selection of exchange sites and docking interactions, these approaches are well positioned to deliver NRP variants tailored for self-assembly, target selectivity and improved therapeutic indices.

These regulatory engineering approaches, combined with understanding of quorum sensing control mechanisms and the development of dual-target screening platforms that map synergistic interactions between multiple regulatory genes, provide powerful strategies for awakening cryptic NRP gene clusters and optimizing production titers in industrial applications.

### 7.4. Synthetic Approaches

#### 7.4.1. Type S NRPS Systems

The development of type S NRPS systems represents a paradigm shift in NRPS engineering. These artificial synthetic NRPS types allow the application of biocombinatorial approaches to the parallel creation of high-throughput NRP libraries. Type S NRPSs utilize synthetic zippers (SZs) that post-translationally restore full-length biosynthetic capability, enabling the application of true biocombinatorial approaches to the development of natural product-like NRP libraries [131]. Optimization efforts have achieved impressive success: titers increased up to 55-fold compared to unoptimized counterparts, allowing production levels to be restored to wild-type levels and even higher. This system allows the creation of two- and three-component NRPS libraries, where each element consists of two or three type S subunits that assemble post-translationally in vivo [131].

#### 7.4.2. Heterologous and Cell-Free Expression

Recent advances in high-throughput engineering utilize yeast surface display systems for screening NRPS modules. By displaying full-length NRPS modules on the surface of yeast cells, condensation activity can be directly linked to fluorescent readouts, allowing the screening of libraries containing up to 108 distinct variants via flow cytometry. This approach achieved a more than 40-fold increase in fatty acid acylation after a single round of mutagenesis and screening [132]. Cell-free protein synthesis platforms offer another powerful tool for NRPS engineering. These systems enable rapid prototyping of engineered NRPS variants without the complexities of cellular expression. Cell-free approaches have been successfully applied to reconstitute complete NRPS pathways and achieve yields comparable to native producers [133].

#### 7.4.3. Hybrid Synthases

The development of hybrid NRPS-PKS assembly lines represents a particularly challenging but promising area. Studies on thalassospiramide biosynthesis have revealed unprecedented biosynthetic patterns involving intermodular substrate activation, module skipping, and reverse chain extension. These systems demonstrate that growing peptide chains can be flexibly passed back to previous modules in a substrate-dependent manner [134]. The work of Präve et al. describes the construction of an NRPS/PKS hybrid via the assembly of various megasynthetase fragments to obtain a modified syrbactin derivative with a complex peptide-polyketide structure acting as a proteasome inhibitor. The engineered product retained the inhibitory activity of the syrbactin class but, due to rational modifications, inhibited immunoproteasomes most strongly [135]. Hybrid NRPS-PKS systems represent frontier targets for engineering complex natural product architectures. These systems require coordination between different catalytic mechanisms and present unique engineering challenges. Successful approaches have focused on interface optimization between NRPS and PKS modules to ensure efficient intermediate transfer. Pass-back chain extension mechanisms in hybrid systems offer opportunities for engineering iterative biosynthetic processes. Understanding these mechanisms enables design of systems where growing chains can be processed multiple times by upstream modules to build complex structural features.

#### 7.4.4. Ribosomal Peptide Emulation

RiPP (ribosomally synthesized and post-translationally modified peptide) biosynthetic machinery can be engineered to emulate NRPS products. This approach offers advantages in terms of genetic tractability and expression efficiency while accessing similar chemical space to traditional NRPs [58].

#### 7.4.5. Modular Biosynthetic Platforms

Standardized biological parts for NRPS engineering are being developed to enable plug-and-play construction of novel biosynthetic pathways. These include characterized promoters, ribosome binding sites, and standardized domain boundaries that facilitate predictable engineering outcomes [114]. Golden Gate assembly methods have been developed for efficient construction of engineered NRPS systems. These approaches enable rapid assembly of complex gene circuits from standardized parts and facilitate high-throughput construction of NRPS variants [136].

#### 7.4.6. Computational Design and Prediction

Structure-based design approaches utilize high-resolution crystal structures of NRPS domains to predict successful engineering targets. Computational analysis of substrate-binding pockets, domain interfaces, and conformational dynamics guides rational mutagenesis strategies [137]. Machine learning approaches are increasingly being applied to predict NRPS substrate specificity and guide engineering efforts. These methods can identify subtle sequence-structure relationships that govern domain function and suggest beneficial mutations for expanding substrate scope [103].

### 7.5. Limitations and Optimization Strategies

Despite significant achievements, a number of challenges remain in NRPS engineering. Protein–protein interactions between domains, especially involving condensation domains, represent a significant factor that can lead to reduced product yields. Disruption of non-covalent interactions between thiolation and condensation domains usually affects multimodular exchanges [138]. Current research on optimization strategies focuses on identifying optimal splicing sites for module exchange. The development of exchangeable units, such as XU (ATC) and XUC (C_acceptor-AT-C_donor), represents progress in addressing fusion compatibility issues. However, experimental results indicate that fusing units from different genera can lead to reduced yields, highlighting the need for further optimization [139]. The diversity of engineering approaches available for NRPS systems reflects the sophisticated molecular machinery involved in non-ribosomal peptide biosynthesis. By targeting different aspects of the biosynthetic mechanism—from individual domain active sites to protein–protein interactions to trans-acting enzymes—researchers can systematically modify NRP structures and properties. Thus, the engineering of new non-ribosomal peptides has evolved from a theoretical possibility into a practical reality. The integration of evolutionary insights, computational tools, and high-throughput screening platforms continues to expand the capabilities for rational NRPS design.

## 8. Self-Assembly Potential of NRPs

NRPs can be seen as a bridge between traditional antibiotics and modern peptide nanotechnology, offering new avenues to overcome resistance. The basic principles governing self-assembly of antimicrobial peptide nanocomplexes involve amphiphilicity characterized by critical micelle concentration, environmental responsiveness and stimuli-triggered assembly, minimization of hemolysis and cytotoxicity toward non-target eukaryotic cells, and proteolytic resistance [140,141]. These principles guide the rational design of therapeutic peptides that spontaneously organize into nanostructures with potent antimicrobial activity while maintaining biosafety.

### 8.1. Self-Assembly Peptide Engineering Basic Principles

#### 8.1.1. Molecular Design Strategies for Self-Assembling Antimicrobials

Rational design of self-assembling antimicrobial peptides requires balancing antimicrobial efficacy with assembly propensity, proteolytic stability, and selective cytotoxicity. Amphiphilic topology segregates hydrophobic and hydrophilic residues into distinct domains, promoting interfacial activity and self-organization [142,143]. Common architectures include facial amphiphiles with segregated α-helical “faces” that orient hydrophilic and hydrophobic residues on opposite sides upon membrane contact [144,145], linear amphiphiles containing hydrophobic cores with charged ends, and block amphiphiles with discrete hydrophobic and hydrophilic segments. Secondary structure propensity is engineered through sequence patterns that favor β-sheets via alternating hydrophobic/hydrophilic residues or aromatic stacking interactions [146]. Strategic placement of aromatic phenylalanine, tryptophan, or tyrosine residues enhances π-π stacking interactions that stabilize β-sheet nanofiber formation along the fiber axis [146,147]. Net positive charge optimizes bacterial membrane binding through electrostatic attraction to anionic phospholipids, with peptide binding correlating directly with membrane surface charge density [148,149]. Non-canonical modifications incorporating D-amino acids, N-methylation, or β-amino acids enhance proteolytic resistance without abolishing self-assembly capability [150,151].

Peptide amphiphiles typically comprise three functional regions: a hydrophobic tail or core formed by alkyl chains, aromatic groups, or hydrophobic peptide sequences that drives aggregation; a structural peptide segment that promotes secondary structure through hydrogen bonding; and a hydrophilic head or corona containing charged residues that provide aqueous solubility and antimicrobial function [152]. The balance between hydrophobic and hydrophilic components, quantified by the packing parameter, determines the resulting morphology [153]. Molecular engineering variables including alkyl chain length, peptide sequence, charge distribution, and aromatic content can be systematically tuned to achieve desired nanostructures [153,154]. Introduction of cohesive β-sheet-forming amino acids near the hydrophobic core favors nanofiber formation over spherical micelles through hydrogen bond alignment along the long axis of the fiber [153,155].

#### 8.1.2. Critical Micelle Concentration and Assembly Thresholds

Self-assembly occurs above the critical micelle concentration (CMC), below which peptides exist as dispersed monomers and above which they form thermodynamically stable assemblies. Antimicrobial peptide amphiphiles typically exhibit CMC values ranging from low micromolar to millimolar concentrations, depending on hydrophobic tail length and ionic strength [156]. For example, amphiphilic Cardin antimicrobial peptide (ACA-PA) displays a CMC at 45 μM, above which it self-assembles into cylindrical supramolecular structures [156]. Fluorescence-based methods enable label-free CMC determination through aggregation-induced emission of peptide amide bonds, revealing that fluorescence CMC occurs at approximately 60 μM for various peptide amphiphiles while true CMC measured by dye partitioning methods is typically an order of magnitude lower at 1–2 μM [157]. The relationship between CMC and antimicrobial activity is crucial for therapeutic applications: antibacterial potency of ACA-PA is remarkably enhanced at concentrations above the CMC against Gram-negative bacteria, demonstrating that self-assembled structures exhibit superior antimicrobial effects compared to monomeric forms [156]. Antibiotic-conjugated peptide amphiphiles such as CPFx-2 exhibit CMC values of 194.99 μM, with enhanced antimicrobial efficacy attributed to synergistic mechanisms between peptide membrane-disruptive action and drug intracellular activity [158].

#### 8.1.3. Environmental Responsiveness and Stimuli-Triggered Assembly

pH-responsive systems exploit ionizable residues including glutamic acid, aspartic acid, histidine, and lysine, whose protonation states change with environmental pH [159]. Histidine residues with pKa near 6 enable charge status changes at physiologically relevant pH values, creating smart systems that assemble or disassemble in response to local pH changes associated with infection sites [160]. For example, peptides containing lysine and arginine residues remain in unimeric states at neutral pH due to electrostatic repulsion but undergo pH-triggered self-assembly into nanofibers when pH increases to 9.4, driven by reduced electrostatic repulsion and increased hydrophobic interactions [159]. pH-responsive antimicrobial peptides incorporating non-natural ionic amino acids exhibit acidity-triggered antimicrobial activity, demonstrating potential for combating infections in pathologically acidic environments [160].

Enzyme-responsive assemblies incorporate cleavage sites for bacterial proteases, enabling on-demand disassembly and drug release specifically at infection sites [161]. Bio-inspired biomaterial coatings with enzyme-sensitive sequences allow triggered release of antimicrobial peptides, achieving reduction in bacterial growth after 4 h of incubation upon enzymatic release [161]. This specificity provides targeted drug release that reduces off-target toxicity while bacterial enzyme upregulation in infected tissues ensures robust activation.

Metal ions, particularly calcium, trigger conformational changes in peptides containing carboxylate-rich sequences [140]. The clinically approved lipopeptide daptomycin exemplifies ion-responsive antimicrobial action: calcium ions neutralize negative charges, triggering oligomerization into functional membrane-disrupting complexes [64,162]. Daptomycin forms micelles of 14–16 monomers at a 2:3 calcium/peptide ratio, undergoing conformational changes that increase amphipathicity and facilitate insertion into phosphatidylglycerol-rich bacterial membranes [162]. Cryo-TEM imaging confirms that daptomycin forms spherical micellar structures only in the presence of both calcium ions and potassium chloride [64]. Daptomycin specifically interacts with undecaprenyl-coupled cell wall precursors (lipid II) in the presence of anionic phospholipid phosphatidylglycerol by forming a tripartite complex, revealing calcium-dependent target specificity [163].

#### 8.1.4. Balancing Antimicrobial Potency with Biosafety

Achieving potent antimicrobial activity while minimizing hemolysis and cytotoxicity represents a persistent challenge [164]. The selectivity index (SI), calculated as the ratio of 50% hemolytic concentration (HC_50_) or 50% cytotoxic concentration (IC_50_) to minimum inhibitory concentration (MIC), quantifies cell selectivity, with higher SI values indicating greater selectivity toward bacterial over mammalian membranes [165,166]. Self-assembly offers multiple strategies to improve the therapeutic window. Structural masking within nanofiber cores reduces non-selective mammalian membrane interactions while preserving bacterial membrane binding through electrostatic interactions [141]. Stimuli-responsive activation designs peptides that remain inactive during circulation and activate only at infection sites via pH, enzymes, or bacterial metabolites, limiting systemic toxicity [167]. Self-assembled peptide nanostructures demonstrate improved hemocompatibility, with PEGylated peptide nanofibers showing increased viability of red blood cells upon treatment compared to non-assembled peptides [168].

#### 8.1.5. Proteolytic Stability Enhancement

Proteolytic degradation represents a major limitation for peptide therapeutics, with serum half-lives often measured in minutes. Self-assembly enhances proteolytic resistance through multiple mechanisms. Peptides embedded in nanofiber cores or micelle interiors exhibit reduced accessibility to proteases [141]. β-sheet conformations in assembled states are inherently more protease-resistant than random coils. The self-assembled peptide Nhar forms long nanofibers through π-π stacking interactions and cation-π interactions, creating amyloid-like fibril structures that protect against proteolytic degradation [141]. Large self-assembled structures exceeding 50 nm cannot access protease active sites designed for monomeric substrates [141]. Self-assembled nanopeptides F3FT and N3FT maintain antibacterial activity after incubation with serum, likely due to steric hindrance preventing hydrolysis by proteases [140]. Nanoengineered self-assembling peptides bearing GHK sequence show improved stability and bioactivity compared to the free tripeptide, which suffers fast proteolytic cleavage in body fluids [169].

### 8.2. Nanostructure Morphologies and Formation Mechanisms

Nanofibers represent the most common self-assembled morphology for antimicrobial peptides, typically spanning diameters of 4–100 nm and lengths reaching micrometers. Their formation is driven by β-sheet secondary structures that laterally associate peptide strands into extended ribbons [170,171]. Chen and colleagues developed self-assembled antimicrobial nanofibers by conjugating the natural peptide melittin to a β-sheet-forming scaffold of alternating glutamine and leucine residues. This co-assembly displayed melittin at the solvent interface, restricting hydrophobic residues and reducing cytotoxicity toward mammalian cells while preserving strong bacterial membrane disruption [170]. Transmission electron microscopy of peptide nanofibers reveals morphological diversity: β-sheet-forming peptides typically produce nanofibers with diameters around 4–5 nm [172,173] while Lanreotide-based peptide nanotubes demonstrate controlled diameters ranging from 10 to 36 nm through precise amino acid substitutions [174]. Yu et al. reported proteolytic-resistant peptide nanofibers that retain antimicrobial activity even under extreme protease exposure (10 mg/mL), employing a trap-and-kill mechanism in which nanofiber networks physically entrap bacteria while inducing bacterial death through membrane disruption [141].

Spherical micelles emerge when amphiphilic peptides adopt high curvature conformations, typically through short hydrophobic tails and bulky charged head groups [152,175]. The hydrophobic core sequesters alkyl chains and aromatic groups, whereas the peptide corona provides aqueous solubility and antimicrobial function [176]. Formation kinetics involve rapid hydrophobic collapse on nanosecond-to-microsecond scales, followed by slower structural equilibration over milliseconds to seconds, with water cage formation and breakage near hydrophobic groups controlling fusion dynamics and aggregation [177]. Coarse-grained simulations show that multiple small micelles form simultaneously before coalescing or transitioning based on interpeptide interactions [178].

Peptide hydrogels consist of three-dimensional networks of self-assembled nanofibers that entrap water, creating viscoelastic materials suitable for injectable formulations and wound dressings [179]. Gelation requires concentrations well above the critical micelle concentration, where nanofibers entangle into percolating networks. These hydrogels exhibit shear-thinning behavior, flowing under applied stress and rapidly recovering gel properties upon stress removal, which enables syringe injection and in situ gelation critical for minimally invasive administration [180,181]. Controlled release from peptide hydrogels often follows biphasic kinetics: an initial burst release caused by protein molecules at or near the solvent-hydrogel interface, followed by sustained release governed by drug–hydrogel interactions [182,183]. Vu et al. confirmed that protein cargo release from peptide hydrogels depends on network stability, cargo mobility, and electrostatic interactions, with controlled release extending beyond 72 h [184].

Peptide vesicles form closed bilayer structures of 50–500 nm diameter when packing parameters approach one-half, mimicking biological membranes and encapsulating both hydrophilic and hydrophobic cargo. Antimicrobial peptide vesicles demonstrate membrane disruption capabilities through peptide binding, membrane thinning, and vesicle budding at high peptide-to-lipid ratios. At peptide-to-lipid ratios exceeding 0.10, antimicrobial peptides induce vesicle protrusion and budding through increased membrane curvature and asymmetric stress on the outer leaflet [185].

### 8.3. NRPs Potential in Self-Assembly

As already mentioned, NRPs inherently possess several properties that are desirable targets for peptide nanocomplexes, and therefore can become their reliable functional foundation.

First, many NRPs are amphiphilic. The inherent amphiphilicity of these NRPs—combining hydrophobic lipid tails with charged or polar peptide heads—provides the thermodynamic driving force for spontaneous self-assembly into functional nanostructures that enhance local antimicrobial concentration at bacterial interfaces and enable stimuli-responsive behavior, making them superior templates for designing next-generation antimicrobial peptide nanocomposites.

Several well-characterized amphiphilic non-ribosomal peptides (NRPs) demonstrate self-assembly properties that make them suitable for constructing antimicrobial nanostructures. Classic examples include the lipopeptides daptomycin, surfactin, polymyxins, fengycin, and mycosubtilin, all of which feature hydrophobic fatty acid tails linked to hydrophilic peptide heads, enabling spontaneous formation of micelles, nanofibers, and vesicles in aqueous environments [186]. Daptomycin—a clinically approved antibiotic—self-assembles into spherical micelles composed of 14–16 monomers in the presence of calcium ions, with amphiphilicity crucial for its aggregation and membrane targeting [64]. Surfactin, produced by *Bacillus* species, forms direct and reverse micelles above the critical micelle concentration and displays transition between spherical and elongated structures depending on concentration and ionic conditions [187]. Fengycin and mycosubtilin, also *Bacillus*-derived, exhibit strong membrane-disrupting activity through amphiphilic-driven self-assembly that enhances their antimicrobial and antibiofilm potency [62].

Polymyxins represent another important class of amphiphilic NRPs, with polymyxin B and colistin (polymyxin E) forming cationic lipopeptide micelles that bind to the anionic lipopolysaccharide layer of Gram-negative bacterial outer membranes through electrostatic interactions while inserting their fatty acid chains into the lipid bilayer [188,189].

Second, it is worth noting the second feature: their mechanism of action closely mirrors that of ribosomally synthesized antimicrobial peptides (AMPs), as both classes frequently disrupt bacterial membranes, causing pore formation or depolarization and ultimately leading to cell death [190,191]. Because membrane damage is a universal and potent antimicrobial strategy, nanocomposites constructed from NRPs can be engineered to serve similar antimicrobial roles as ribosomal AMPs, functioning as agents for bacterial eradication or membrane-targeted therapy [192,193].

Third, many NRPs possess a tendency to self-assemble and form nanostructures, not only due to their amphiphilicity described above. Many non-ribosomal peptides exhibit intrinsic self-assembly tendencies beyond amphiphilicity, driven by multiple molecular interactions that enable nanostructure formation. Hydrogen bonding networks enable β-sheet formation in both linear and cyclic peptides—for instance, cyclic peptides composed of alternating D- and L-amino acids self-assemble into nanotubes through parallel or antiparallel β-sheet stacking [194], while the inclusion of non-coded amino acids ensures the formation of superhelical large, ordered structures [195].

Fourth, NRPs possess enhanced proteolytic resistance compared to linear ribosomal peptides, a property conferred by multiple structural features that prevent recognition and cleavage by proteases. Key resistant NRPs include the antibiotics daptomycin, polymyxin B, vancomycin, and teicoplanin, as well as the immunosuppressant cyclosporine [57,58]. These modifications are enzymatically installed during biosynthesis by non-ribosomal peptide synthetases (NRPSs) and include the following:

(1) D-Amino Acids: These stereoisomers alter substrate recognition by proteases. D-amino acid incorporation provides substantial proteolytic stability, with D-peptides showing no degradation after 24 h in human serum [58].
(2) N-Methylation: N-methyltransferase domains methylate backbone amides, disrupting hydrogen bonding networks required for protease substrate recognition. In cyclosporine, seven of eleven residues are N-methylated, creating steric hindrance that prevents proteolytic attack [196]. N-methylation at specific positions increases half-life from 4 h to over 24 h in the presence of chymotrypsin. This modification eliminates hydrogen bond donors, reducing water interactions and protease binding [57,197].
(3) β-Amino Acids and Non-Proteinogenic Residues: Incorporation of β-amino acids and δ-amino acids (such as 4-aminocyclohexanecarboxylic acid) increases proteolytic resistance by altering backbone geometry and reducing conformational flexibility [195].
(4) Macrocyclization: Cyclization protects peptide termini from exopeptidases and imposes conformational rigidity that reduces accessibility by endopeptidases [198].
(5) Fatty Acylation: Lipopeptides like daptomycin contain lipid moieties that anchor them to membranes, reducing solvent exposure and protease access [198].

These modifications obviously can directly facilitate self-assembly.

Proteolytic resistance ensures that self-assembling NRP nanostructures persist in biological fluids. Yu et al. demonstrated that D-amino-acid-containing peptide nanofibers maintain structural integrity in vivo while trapping and killing bacteria, a functionality lost in protease-sensitive analogs [141]. D-amino acids and N-methylations reduce conformational entropy, promoting ordered β-sheet formation and π-π stacking interactions that drive fibrillization. In Nhar peptide nanofibers, D-residues and aromatic stacking create stable amyloid-like structures with 67.7% β-sheet content [141]. Lipidation and unbiogenic amino acids enable precise hydrophobic-hydrophilic segregation, directing supramolecular assembly [58].

NRPS engineering can install these modifications combinatorially. Muangkaew et al. note that hybrid E/C domains in *Pseudomonas* lipopeptides epimerize and condense simultaneously, producing peptides with dual resistance and assembly propensity [199].

**Figure 2:**
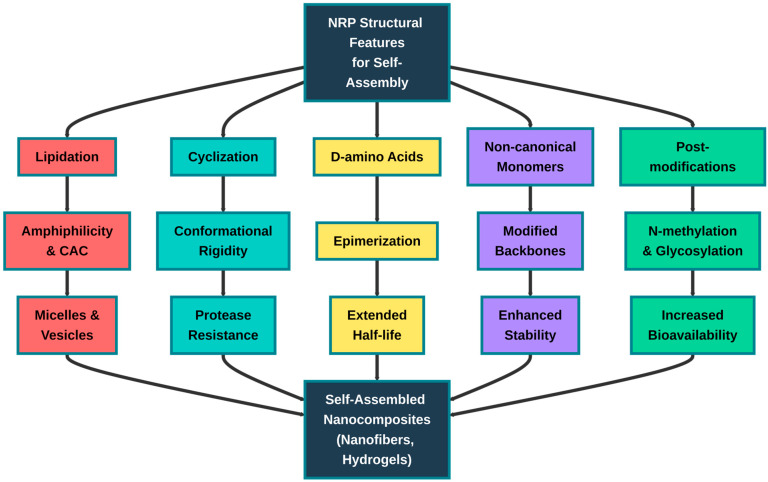
NRP structural features for self-assembly.

The main properties of NRPs, which allow them to be considered as a substrate for self-assembling complexes, are summarized in Figure 2.

Thus, the same NRPS-derived modifications that confer protease resistance—D-amino acids, N-methylation, cyclization, and lipidation—simultaneously encode the stereochemical and physical constraints necessary for controlled self-assembly into functional complexes.

### 8.4. NRP-Based Combinatory Strategies

Based on the structural diversity and unique biosynthetic mechanisms of non-ribosomal peptides (NRPs), several rational strategies can be proposed for their application in self-assembled antimicrobial nanocomplexes.

#### 8.4.1. NRP-Based Frameworks for Hybrid Complexes

NRPs produced by bacilli, particularly polymyxins and gramicidin S, exhibit significant potential as structural frameworks for hybrid antimicrobial nanocomplexes. Polymyxin B (PMB) demonstrates high binding affinity to lipopolysaccharide (LPS) on Gram-negative bacterial membranes and inhibits Toll-like receptor 4 (TLR4) signaling, thereby down-regulating acute inflammatory responses [200]. However, systemic toxicity limits clinical applications. To address this, nanoparticle carriers can modify PMB biodistribution and reduce toxicity to critical organs while preserving antibacterial activity [200]. Specifically, self-assembled tannic acid/Fe^3+^ nanoparticles carrying PMB on their surface enable selective presentation of PMB to endotoxins and bacteria while limiting direct exposure to mammalian cell membranes [200]. These nanoparticulate formulations maintain antimicrobial activity against clinically isolated Gram-negative pathogens including *E. coli*, *K. pneumoniae*, and *P. aeruginosa*, with only two- to fourfold increases in minimum inhibitory concentrations compared to free PMB [200]. Negatively charged nanodiscs have also been developed to reduce polymyxin B toxicity through controlled presentation of PMB on nanoparticle surfaces [201]. These findings support the development of micelle-based delivery systems loaded with bacilli-derived NRPs to improve stability and reduce toxicity against Gram-negative infections.

#### 8.4.2. Stimulus-Responsive Self-Assembly

The incorporation of stimulus-responsive motifs into antimicrobial peptide systems enables controlled self-assembly of nanostructures. pH-responsive antimicrobial peptides demonstrate this principle through environment-dependent structural transitions. Synthetic peptides incorporating phenylalanine form hydrogels that maintain antimicrobial activity with pH-dependent gelation properties [159]. Circular dichroism spectroscopy reveals that increased phenylalanine content enhances α-helical structure formation, correlating with improved gel-forming abilities. Transmission electron microscopy demonstrates distinct nanostructure variations under different pH levels, with fibrous network configurations forming at high pH and nanoparticle assembly at low pH [159]. Similarly, lipopeptide self-assembly shows pH-dependent β-sheet formation, where thioflavin T fluorescence confirms β-sheet structures in extended nanostructures at pH 8 that are absent at pH 4.6 [65]. This pH-triggered structural transition from disordered/random coil to β-sheet secondary structure enables controlled nanostructure formation [65]. pH-responsive co-assembled peptide hydrogels demonstrate antibacterial activity against methicillin-resistant *Staphylococcus aureus* (MRSA) and promote wound healing [202]. These mechanisms validate the strategy of engineering pH-activated β-sheet nanostructures for stimulus-responsive antimicrobial activity.

#### 8.4.3. Hybrid Ribosomal–Non-Ribosomal Peptide Constructs

The creation of hybrid peptides combining NRPs with ribosomally synthesized antimicrobial peptides (AMPs) represents a promising approach for enhanced antimicrobial efficacy. While native RiPPs cannot explore amino acids beyond the canonical 20 proteinogenic amino acids, genetic code expansion enables insertion of non-canonical amino acids (ncAAs) into growing peptide chains, achieving high chemical diversity at low genetic cost [203]. This methodology allows the production of lantibiotic derivatives with enhanced proteolytic resistance and increased bioavailability. Nisin, a model lantibiotic, has been engineered through residue alterations incorporating Nε-Boc-L-lysine (BocK) at specific positions, producing bioactive nisin variants with non-canonical amino acids [203,204]. Hybrid antibiotic design targeting the bacterial ribosome demonstrates that rational combination of different antimicrobial moieties can restore or enhance antibacterial efficacy against resistant strains [205]. Specifically, an azithromycin–tedizolid hybrid exhibited activity against a diverse panel of multidrug-resistant Gram-positive bacteria including strains with macrolide and oxazolidinone resistance mechanisms [205]. The potential for peptide fusions combining NRP scaffolds with ribosomal AMPs warrants investigation for dual membrane disruption mechanisms and immunomodulatory effects.

#### 8.4.4. Antimicrobial Synergy and Biofilm Disruption

NRPs such as surfactins exhibit potent biofilm disruption capabilities that synergize with conventional antibiotics and nanostructured AMPs. Surfactant polymer dressings demonstrate synergistic inhibition of biofilm viability when combined with antibiotics against *P. aeruginosa* and methicillin-resistant *S. aureus* [206]. This synergy reflects improved antibiotic penetration and accessibility to bacterial targets within biofilms, mediated by surfactant-induced disruption of extracellular polymeric substance matrix structure [206]. Sophorolipid biosurfactants cause catastrophic disruption of *P. aeruginosa* biofilms at concentrations significantly below their critical micelle concentration, weakening the EPS matrix and leading to surface detachment [207]. Importantly, sophorolipids act cooperatively with sodium dodecyl sulfate, disrupting nearly 100% of biofilms when used in combination, compared to approximately 70% disruption by sophorolipids alone [207]. Surfactin demonstrates antibacterial activity through membrane disruption and inhibits penicillinase, suggesting potential synergy with β-lactam antibiotics [208]. These mechanisms support the analysis of NRPs as biofilm-disrupting agents that enhance the efficacy of nanostructured AMPs against resistant pathogens.

#### 8.4.5. NRP–Hydrogel Composite Materials

The integration of NRPs into hydrogel matrices creates composite materials suitable for wound dressings and implant coatings. pH-responsive self-assembled peptide hydrogels loaded with antimicrobial agents demonstrate controlled release properties and antibacterial activity [209]. Surfactant-based dressings exhibit potent anti-biofilm properties through direct effects on bacterial cell viability and disruption of inter-bacterial physical interactions, inhibiting quorum sensing pathways [206]. The inherent anti-adhesive properties of surfactin produced by bacilli can be leveraged in composite hydrogel formulations for wound care applications. pH-sensitive supramolecular self-assembled peptide hydrogels provide sustained release profiles and enhanced antimicrobial efficacy [210]. These composite materials exploit both the antimicrobial activity of NRPs and the structural benefits of hydrogel matrices for biomedical applications.

#### 8.4.6. Scalability Through Fermentation Optimization

Addressing production scalability requires systematic optimization of *Bacillus* fermentation parameters for NRP synthesis. Statistical optimization using response surface methodology enables significant improvements in bioproduction yields. For *B. subtilis*, numerical optimization using Plackett–Burman design followed by Box–Behnken design identified optimal conditions including specific carbon and nitrogen sources, temperature, pH, and aeration parameters, resulting in substantial increases in surfactin production [211,212]. Combinatorial metabolic engineering of *B. subtilis* ATCC 21332, including enhanced nitrate reduction, fatty acid hydroxylation, rational transporter engineering, and feeding strategies, yielded 14.4 g/L of surfactin with productivity of 0.6 g/L/h [211]. These optimization strategies reduce time and costs while providing precise control over critical production parameters, enabling mass production of NRP nanocomplexes for therapeutic applications.

**Figure 3:**
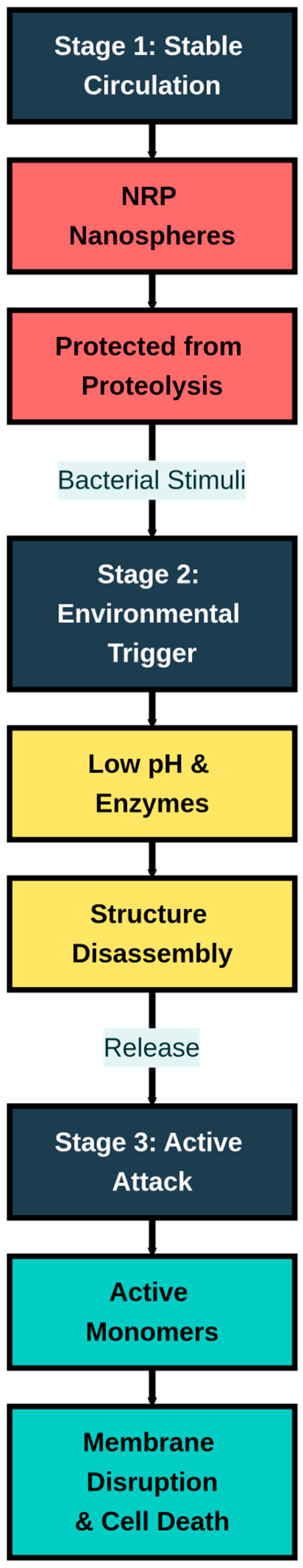
Stages of NRP-based complex action.

The main stages of NRP-based complex potential action are summarized in Figure 3.

## 9. Conclusions

The escalating crisis of antimicrobial resistance necessitates the exploration of therapeutic platforms that go beyond conventional small-molecule antibiotics and monomeric antimicrobial peptides. While ribosomally synthesized AMPs have been the primary focus of self-assembling nanostructure research, this review underscores the underutilized potential of non-ribosomal peptides (NRPs) as engineerable scaffolds for such systems. Intrinsic NRP features—including D-amino acids, macrocyclization, N-methylation, lipidation and complex post-assembly tailoring—naturally confer proteolytic stability, amphiphilicity and membrane activity that typically have to be artificially introduced into ribosomal peptides.

A central innovative aspect highlighted here is the integration of NRPS-level genetic engineering with materials-driven control of peptide self-assembly. Rather than considering NRPs solely as individual antibiotics, we outline how domain and module engineering, CRISPR-based editing of biosynthetic gene clusters, and synthetic type S NRPS platforms can be used to generate NRP variants that are predisposed to form defined supramolecular architectures. These engineered NRPs, together with NRP-inspired motifs, can be embedded into nanofibers, micelles, hydrogels and vesicles to yield stimuli-responsive antimicrobial complexes with enhanced local bioavailability, reduced systemic toxicity and the ability to circumvent existing resistance mechanisms through membrane-disrupting and multimodal modes of action.

Looking ahead, several research directions appear particularly promising for advancing NRP-based self-assembling antimicrobial complexes. First, evolution-guided and high-throughput NRPS engineering, supported by computational and machine-learning tools, should expand the accessible NRP chemical space and enable more predictable installation of self-assembly-promoting and stimuli-responsive motifs. Second, combining NRPs with complementary components—such as ribosomal AMPs, synthetic polymers, inorganic carriers or responsive linkers—is expected to generate hybrid “smart” nanocomposites that couple membrane disruption with controlled release, immunomodulation or biofilm penetration. Third, systematic in vivo evaluation in clinically relevant infection and biofilm models, together with rigorous assessment of hemocompatibility, cytotoxicity and immune responses, will be essential for translating these concepts into realistic therapeutic candidates. By bridging natural product biosynthesis with supramolecular nanotechnology, engineered self-assembling NRP complexes have the potential to contribute substantially to the next generation of antimicrobial therapies.

## Figures and Tables

**Table 1:** Comparative physicochemical and pharmacological properties of canonical human antimicrobial peptides (LL-37, HNP-1) and typical non-ribosomal peptides (NRPs).

Parameter	LL-37 (Cathelicidin)	HNP-1 (α-Defensin)	Typical NRP (Cyclic Lipopeptide, e.g., Daptomycin/Polymyxin)
Origin	Ribosomally synthesized pro-protein hCAP-18, released by proteolysis during inflammation.	Ribosomally synthesized precursor, processed and stored in neutrophil granules.	Non-ribosomal product of modular NRPS with cyclization and non-proteinogenic residues.
Length/structure	37 residues; linear amphipathic α-helix; no disulfide bonds; prone to oligomerization.	29–30 residues; compact β-sheet stabilized by three disulfide bonds; dimer/oligomer in membranes.	Typically cyclic or lipo-/glycopeptide with rigid conformation and pre-organized amphiphilicity.
Charge/amphiphilicity	Strongly cationic (≈+6), facial helical amphiphilicity, high affinity for anionic membranes.	Cationic β-sheet with defined hydrophobic and charged surface patches.	High positive charge combined with fatty-acid tail and/or extended hydrophobic segments.
Membrane mechanism	Carpet-like adsorption and transient channel formation; possible translocation to intracellular targets.	Dimer-pore mechanism with cationic pores disrupting ion homeostasis.	Stable pore or mixed-micelle formation with specific lipids (e.g., LPS, phosphatidylglycerol).
Immunomodulation	Potent chemotactic and immunomodulatory activities; modulates TLR signaling and cytokine profiles.	Regulates inflammation and chemoattraction; function tuned by post-translational modifications.	Immunomodulatory effects reported but often compound-specific and less systematically characterized.
Proteolytic stability	Rapidly degraded in serum and wound exudate; stability improved by cyclized/modified derivatives.	Relatively protease-resistant due to disulfide-stabilized β-sheet, but still degradable in inflamed tissues.	Generally high proteolytic stability owing to macrocyclization and non-standard residues.
Cytotoxicity/hemolysis	Hemolytic and cytotoxic at low-to-mid µM concentrations; limits systemic use.	Host–cell damage at high concentrations; modifications can reduce membrane toxicity.	Significant toxicity for some (e.g., nephro-/neurotoxicity of polymyxins); others more favorable but still monitored.
Pharmacokinetics	Short-lived peptide acting mainly at local inflammatory or epithelial sites.	Locally acting peptide released from neutrophils; transient in body fluids.	Approved NRPs display plasma half-lives of several hours and defined tissue distribution suitable for systemic therapy.

## Data Availability

No new data were created or analyzed in this study. Data sharing is not applicable to this article.

## Abbreviations

The following abbreviations are used in this manuscript:

A-domain	Adenylation domain
ACAT	Acyl-CoA Cholesterol Acyltransferase
Aib	2-Aminoisobutyric acid
AL-domain	Acyl ligase domain
AMP	Antimicrobial Peptide(s)
ATP	Adenosine triphosphate
BocK	N-Boc-L-lysine
C-domain	Condensation domain
CAC	Critical Aggregation Concentration
Combi-OGAB	Combinatorial Ordered Gene Assembly in Bacillus
CRISPR-Cas9	Clustered Regularly Interspaced Short Palindromic Repeats-CRISPR-associated protein 9
Cs-domain	Starter condensation domain
Cy-domain	Cyclization (Heterocyclization) domain
Dab	2,4-Diaminobutyric acid
E-domain	Epimerization domain
EPS	Extracellular Polymeric Substance
FAAL	Fatty acyl-AMP ligase
HPG	Hydroxy-phenyl glycine
IL-2	Interleukin-2
LPS	Lipopolysaccharide
MRSA	Methicillin-Resistant Staphylococcus aureus
MT-domain	Methylation domain
mTOR	Mammalian Target of Rapamycin
ncAAs	Non-canonical amino acids
NFAT	Nuclear Factor of Activated T cells
NRP	Non-Ribosomal Peptide(s)
NRPS	Non-Ribosomal Peptide Synthetase(s)
Orn	Ornithine
Ox-domain	Oxidation domain
PCP-domain	Peptidyl Carrier Protein domain
PKS	Polyketide Synthase
PMB	Polymyxin B
PP	4′-Phosphopantetheine
PP1	Protein Phosphatase 1
PP2A	Protein Phosphatase 2A
Ppant	Phosphopantetheine (cofactor)
R-domain	Reductase domain
rm Combi-OGAB	Random mutagenesis Combi-OGAB
SDS	Sodium Dodecyl Sulfate
SEAM-OGAB	Seam Express Assembly Method–Ordered Gene Assembly in Bacillus
T-domain	Thiolation domain
TE-domain	Thioesterase domain
TEII	Type II thioesterase
TLR4	Toll-like Receptor 4
VRE	Vancomycin-Resistant Enterococcus
